# Long-Term Effects of Multimodal Treatment on Psychopathology and Health-Related Quality of Life of Children With Attention Deficit Hyperactivity Disorder

**DOI:** 10.3389/fpsyg.2019.02037

**Published:** 2019-09-24

**Authors:** Szabina Velõ, Ágnes Keresztény, Gyöngyvér Ferenczi-Dallos, Judit Balázs

**Affiliations:** ^1^Doctoral School of Psychology, Institute of Psychology, Eötvös Loránd University, Budapest, Hungary; ^2^Department of Developmental and Clinical Child Psychology, Institute of Psychology, Eötvös Loránd University, Budapest, Hungary; ^3^Vadaskert Foundation, Child Psychiatry Hospital and Outpatient Clinic, Budapest, Hungary; ^4^Bjørknes University College, Oslo, Norway

**Keywords:** ADHD, quality of life, long term, multimodal treatment, comorbid disorders

## Abstract

**Aim:**

The current study aimed to examine the association between long-term (36 months) multimodal (pharmacological and psychological) treatment and psychopathology and health-related quality of life (HRQoL) in children with attention deficit/hyperactivity disorder (ADHD) from the perspectives of both the children and parents.

**Methods:**

The sample consisted of 23 children with ADHD (21 boys, 2 girls, mean age = 13.46 years, *SD* = 2.36) and 23 healthy control children (11 boys, 12 girls, mean age = 12.49 years, *SD* = 1.75). The Mini International Neuropsychiatric Interview for Children and Adolescents (MINI Kid) was applied to measure psychopathology and both parent and self-rated versions of the Inventory for the Measure of the Quality of Life in Children and Adolescents were used to assess HRQoL at baseline and at the 36-month follow-up visit. The ADHD group took part in multimodal (medical and behavioral) therapy. The healthy control group did not get any intervention.

**Results:**

At the baseline, the ADHD group was characterized with higher scores in nine MINI Kid scales and showed lower HRQoL than the control group according to both children and their parents. At the 36-month follow-up visit six scale scores (ADHD, social phobia, oppositional defiance and conduct disorder, major depressive episode, dysthymic disorder) showed statistically significant decreases in the ADHD group, while these scores were constant in the control group. Parent-rated HRQoL was significantly lower in the clinical group at baseline than at the end of the study, but there were no significant changes in the control group. Self-reported changes in HRQoL matched parent-reported changes.

**Interpretation:**

Multimodal therapy is associated with decreased psychopathology and improved HRQoL over the long term.

## Introduction

Attention deficit hyperactivity disorder (ADHD) is a highly prevalent behavioral disorder in childhood and adolescence with early onset (Brown et al., 2001; Polanczyk et al., 2014) and it may persist into adulthood in 30–60% of cases (Scahill and Schwab-Stone, 2000). More than 80% of children with ADHD have at least one comorbid psychiatric disorder and 50% of them are likely to have two (Gillberg et al., 2004). Boys with ADHD have more comorbid externalizing problems and girls with ADHD (especially adolescents) have more comorbid internalizing problems such as depression and anxiety (Rucklidge, 2008). Comorbidity may also play a role in their impairments in social, emotional, and academic functioning (Loe and Feldman, 2007; Wehmeier et al., 2010).

Increased family and parental stress appear in the families of children with ADHD (Theule et al., 2013), which is likely to be an important factor leading parents to seek treatment for their child (Cannon et al., 2009). Effective multimodal treatment exists for the management of ADHD, including parental education, cognitive behavioral therapy, and medication (Sonuga-Barke et al., 2013; Hinshaw et al., 2015; National Institute for Health and Care Excellence, 2018). According to the recommendation of the National Institute of Clinical Excellence (NICE) clinical guideline (National Institute for Health and Care Excellence, 2018), treatment need to be preceded by giving a detailed information about ADHD which underlies the pharmacological and non-pharmacological treatment as well. The information should include the severity of symptoms and impairments, education on the causes of ADHD, treatment plans, and possible outcomes. NICE guideline offers for parents and careers a parent-training program as first-line treatment for children under 5 years with ADHD or for children aged 5 years or over who have ADHD and comorbid symptoms of oppositional defiant disorder or conduct disorder. Parental training has beneficial effects on parenting and on conduct problems which commonly co-occur with ADHD (Daley et al., 2018). According to the NICE guideline (National Institute for Health and Care Excellence, 2018) methylphenidate is recommended as the first line pharmacological treatment for children – aged 5 years or over – with ADHD who need medication. Cortese et al. (2018) compared ADHD medications in terms of efficacy on core ADHD symptoms, clinical global functioning, tolerability, acceptability, and other clinically important outcomes. Evidence from this meta-analysis also supports methylphenidate (in children and adolescents) as the preferred first pharmacological choice for short-term pharmacological treatment of ADHD.

Quality of life (QoL) can be an important outcome measure (Coghill, 2010) for understanding the impact of a mental disorder on patients’ functioning and for assessing the effectiveness of treatment. There is no clear separation between the concept of QoL and the concept of health-related quality of life (HRQoL), the latter being used in the medical field in many publications (Coghill et al., 2009). In this paper, the term of HRQoL will be used. In recent years, HRQoL and its relationship with mental disorders have become important in child and adolescent psychiatry (Coghill et al., 2009). HRQoL is a multidimensional concept that includes the physical, social, and emotional components of health (Danckaerts et al., 2009). Several studies have established that children with ADHD have lower HRQoL than their healthy peers (Danckaerts et al., 2009; Velõ et al., 2013). It has also been emphasized that there is low child–parent agreement in rating HRQoL (Danckaerts et al., 2009; Cortese, 2010; Velõ et al., 2013). Children with ADHD rate their own HRQoL less negatively than their parents do, so the assessment of HRQoL from different perspectives is important. Based on the study of Dallos et al. (2017) lower self-reported HRQoL of children with ADHD was associated with female gender and higher age, furthermore lower HRQoL was related to older age according to the parents’ report. Having established the negative effect of ADHD on HRQoL, there is a growing demand to ascertain the impact of treatment on reducing ADHD symptoms and improving HRQoL. Coghill et al. (2017) and Coghill (2010) have systematically reviewed studies of ADHD medication and HRQoL. These studies revealed a positive short-term (2–24 weeks) effect for pharmacotherapy on HRQoL and on the remission of ADHD symptoms. Flapper and Schoemaker (2008) investigated the impact of the combined diagnoses of developmental coordination disorder and ADHD on HRQOL and the effectiveness of a short-term pharmacological therapy on HRQoL from the child and proxy perspective. They found significant improvements in HRQoL reported both by parents and children after a 4-week period of pharmacological treatment, in addition to an improvement in the symptoms of ADHD and motor functioning. Although the short-term effect for pharmacotherapy on HRQoL and on the remission of ADHD symptoms are well-described, the persistence of these effects in long term (>24 weeks) has been understudied. It is worth mentioning that ADHD is a long-term mental disease (Biederman et al., 2010), so follow-up studies are highly important. Coghill (2010) also highlighted the necessity of a long-term examination and emphasized the urgent need to measure the impact of psychological therapies on their own or in combination with medication on HRQoL in children with ADHD.

Based on all above the current study examines the association between long-term (36 months) multimodal (pharmacological and psychological) treatment and psychopathology (symptoms of both ADHD and comorbid disorders) as well as HRQoL in children and adolescents with ADHD from both the children’s and parents’ perspectives.

Our hypotheses are:

Hypothesis 1. At the end of the study (36 months) children in the clinical group are characterized with less psychopathology (both ADHD symptoms and comorbid psychopathology) than at the baseline.
Hypothesis 2. At the end of the study (36 months) children in the clinical group are characterized with better HRQoL both based on self- and parent’s reports than at the baseline.
Hypothesis 3. Changes in psychopathology and HRQoL are independent from gender and social status.

## Materials and Methods

### Participants

We involved into the study a clinical and a healthy control groups of children. Treatment-naïve children with ADHD were enrolled in the present study from the Vadaskert Child Psychiatric Hospital and Outpatient Clinic, Budapest, Hungary. Definition of treatment-naïve children with ADHD was: children without previous medication or psychotherapy treatment of ADHD. We included all children into the clinical group, whose further combined treatment was administered according to the instructions of their child psychiatrist (therapist for their regular treatment) in the hospital. Addition inclusion criteria were that children had to meet the diagnostic criteria for ADHD according to a structured diagnostic interview (see below) and be between the ages of 6 and 18 years. The exclusion criterion was mental retardation in the medical history. Moreover, we excluded in the follow-up study all children whose child psychiatrist (therapist for their regular treatment) in the hospital did not suggest further pharmacological treatment (i.e., lack of efficacy or due to side effects).

A healthy control population from the same age group was recruited randomly from elementary schools from Budapest and the countryside. The exclusion criteria in the control group were ongoing and/or previous psychological and/or psychiatric treatment. Lack of any psychiatric diagnosis was confirmed by a structured psychiatric interview (see below).

To reveal the role of social background, the groups were sorted into four categories based on the parents’ educational and economic status. The Low1 category of children included those whose parents have completed eight classes or a certificate of professional qualification or maturity and only one parent has an active income. In the Low2 category, parents have the same educational background but both parents have an active income. Children whose parents have a BA or an MA degree and only one parent has an active income are grouped in High1, while children whose parents have a BA or an MA degree and two active incomes are in the High2 group.

This study was approved by the Ethical Committee of the Medical Research Council, Hungary (ETT-TUKEB). The parents of each child and children older than 14 years of age included in this study provided written informed consent after being informed of the nature of the study. No compensation was provided to the participants.

### Measures

#### Psychiatric Symptoms and Diagnoses

To measure psychopathology and diagnoses both in the clinical and healthy control groups, the modified version of the Hungarian Mini International Neuropsychiatric Interview for Children and Adolescents (MINI Kid) 2.0 (Lecubrier et al., 1997; Sheehan et al., 1998, 2010; Balázs et al., 2004) was applied. The MINI Kid is a structured psychiatric interview that assesses 25 DSM-IV child and adolescent psychiatric disorders. The modified version of the MINI Kid assesses all of the psychiatric symptoms of the disorders. The interview is suitable for children aged between 6 and 18 years; the interview was given to children under 13 years of age in the presence of their parent, while those above 13 years of age received the interview on their own. Based on the inter-rater reliability, test–retest reliability, sensitivity, and specificity, the Hungarian version of the MINI Kid proved to be reliable (Balázs et al., 2004). Before analysis, the scores for raw symptoms in each section were converted to a 0–100 scale, with higher scores indicating greater severity of symptoms. In the present study 13 scales were used: ADHD, oppositional defiant disorder, conduct disorder, major depressive episode, dysthymic disorder, (hypo)manic episode, panic disorder, separation anxiety disorder, social phobia, specific phobia, generalized anxiety disorder, tic disorder, and obsessive-compulsive disorder.

#### HRQoL

The Hungarian version of the “Erfassung der Lebensqualität Kindern und Jugendliche” (Measure of QoL for Children and Adolescents; ILK) scale (Kiss et al., 2007; Remschmidt and Mattejat, 2010) was applied to assess HRQoL. The questionnaire measures general HRQoL in six different domains: school, family, peer relations, being alone, somatic health, and mental state. We used both child self-report and a parallel parent-proxy report format. The child version of the scale used facial expressions of emotions (laughing, smiling, neutral, sad, and crying) to evaluate the items. The items were scored on a 5-point Likert scale. Based on the validating study, the Hungarian version of ILK showed adequate internal consistency (Cronbach’s alpha ≥0.6), test–retest reliability (Kappa = 0.67–0.75), and discriminant validity (Kiss et al., 2007). The raw score on each scale was converted to a 0–100 scale, with higher scores indicating a better HRQoL.

### Procedure

At baseline (T1) following informed consent, the children in clinical and healthy control groups were evaluated to determine whether the inclusion criteria were met without any of exclusion criteria. At first, the modified version of the MINI Kid was applied to measure psychopathology and diagnoses both groups. In the case of children under 13 we interviewed the parents and the children together and according to the manual of the MINI Kid questions were directed to the children, but the parents were encouraged to add their opinion and the interviewer made the final decision, while above 13 we interviewed the children without their parents. Next, children and parents completed ILK questionnaires, and parents were asked not to assist their children during this procedure. After the baseline measurements children in the clinical group were medicated with an optimal dose of methylphenidate, which was administered according to the instructions of their child psychiatrist (therapist for their regular treatment). In Hungary both short and long acting methylphenidate are available. Normally children are suggested take the morning dose before going to school. Treatment regime follows the label of methylphenidate. Additionally all children took part in a 2-week group therapy, named “Fészek” program (Fészek is the Hungarian word for “Nest”) (Kis et al., 2017) based on cognitive behavioral therapy at the Vadaskert Child Psychiatric Hospital. “Fészek” program is a small group for school-aged children with ADHD. The aims of the 2-week program are to decrease conduct problems, develop attention abilities, practice learning strategies, and develop self-esteem and social competences. Trainers help to apply household rules and clear limits which are supported by pictures. Household rules focus on behavioral difficulties of children with ADHD (e.g., keeping calm when somebody speak, sitting on my chair, etc.). Trainers also suggest them alternative behaviors which tent to improve children’s social skills. Children with ADHD are motivated by praise, encouragement, and rewards for observing household rules and completing lessons. In the case of non-desirable behavior, trainers remind children for absence of reward. Parents of children with ADHD also take part in a four occasion parent training connecting to “Fészek” program. The aim of the parent training is to teach parents technics and strategies which can help them to manage their children’s difficulties: effective communication and creation of visualized household rules and techniques to facilitate demand behavior (i.e., praise, encouragement, rewarding, redirection, distraction, ignoration, and consequences). Every occasion is interactive and practical and focuses on everyday life problems of families where the child/children has/have ADHD.

During the visits, the optimal dose of medication and behavioral interventions was discussed. Monthly follow-up visits to assess the multimodal treatment were held in the Outpatient Department of Vadaskert Hospital, and a detailed general medical visit was held every 6 months. Control group did not get any intervention. After 36 months (T2), all children from clinical and non-clinical groups were contacted again and asked to participate in a follow-up visit. If a parent did not answer the phone (the parents gave us their number previously), we contacted them two further occasions via phone, moreover we sent them regular mail as well. During the follow-up visit the modified version of the MINI Kid and the ILK questionnaires was completed again.

### Statistical Procedures

Descriptive statistics are reported in the text. The chi-squared test of independence was calculated to examine the relationship between categorical variables with the Fisher exact test for 2 × 2 crosstab (group and gender) and Pearson’s chi-square statistics (group and social status). Phi or Cramer’s *V* was used as the measure of associations. An independent sample *t*-test was conducted to compare the MINI Kid scales and HRQoL for the children or parents in the clinical and control group at T1. Also independent sample *t*-test was used to compare children with ADHD who completed the multimodal treatment with children with ADHD who did not completed the multimodal treatment regarding age, HRQoL, and ADHD symptoms at T1. Differences over time between T1 and T2 according to groups were examined using 2 × 2 mixed analysis of variance controlling for age (ANCOVA) with Group (clinical vs. control) as the between-subject factor and Time (T1 vs. T2) as the within-subject factor to determine the effect of the intervention on the MINI Kid scales and HRQoL according to children or parents. Further 2 × 2 × 2 or 2 × 2 × 4 mixed analysis of variance controlling for age (ANCOVA) was performed for two between-subject factors (2 × 2: Group and Gender or 2 × 4: Group and Social status) and Time (T1 vs. T2) to examine the confounding factors (gender or social status) on the effects of the intervention on the MINI Kid scales and HRQoL according to the children or parents. For effect size, partial eta-squares were calculated. All data analyses were computed with IBM SPSS 25 (IBM Corp, 2017).

## Results

### Participants

The full sample of the ADHD group consisted of 79 children [64 (81%) boys and 15 (19%) girls] at the baseline stage of the investigation. Mean age of children with ADHD was 10.24 years (*SD* = 2.51, range:10–18). The control group was composed of 54 children [31 (57.4%) boys and 23 (42.6%) girls] with a mean age of 9.66 years (*SD* = 1.73, range: 9–16). There was a non-significant difference in age between the clinical and the control group [*t*(129) = -1.574, *p* = 0.118]. The gender and the group revealed a significant relationship [χ^2^(1) = 8.758, *p* = 0.006, Φ = 0.26]. The proportions of boys from the clinical group were 81% (64 boys), it was 57.4% (31 boys) in the control group. The relationship between the social status and the group was significant [χ^2^(3) = 25.421, *p* < 0.001, Cramer’s *V* = 0.45]. The frequencies of the social status categories differed across the group levels.

The follow-up sample consisted of 46 children: 23 children in the clinical group and 23 children in the control group. Table 1 presents the reasons of drop out and the number of participating children at the 36-month follow-up visit. There was no significant difference in age [*t*(44) = -1.576, *p* = 0.123] between the clinical (*M* = 13.46, *SD* = 2.36) and the control group (*M* = 12.49, *SD* = 1.75).

**TABLE 1:** The reasons of drop out and the number of participating children at the 36-month follow-up visit in the clinical and in the control groups.

	**Clinical group (*n*)**	**Control group (*n*)**
At the baseline visit	79	54
Rejected the participation at the 36-month follow-up visit	27	26
Not available at the 36-month follow-up visit	27	5
Non-medicated during 36 months (excluded)	2	0
At the end of the study (at the 36-month follow-up visit)	23	23

**n*, number of children.*

The relationship between gender and group was significant [χ^2^(1) = 10.268, *p* = 0.003, Φ = 0.47]. The proportion of boys from the clinical group was 91.3% (21 boys), whereas the proportion in the control group was 47.8% 11 boys; Table 1. The social status of the groups also showed a statistically significant difference [χ^2^(3) = 12.468, *p* = 0.006, Cramer’s *V* = 0.53]. The frequencies of the High2 and Low2 social statuses differed across group levels.

There was a non-significant difference in age between the treatment completers and the non-completers [*t*(76) = 0.633, *p* = 0.530] in the clinical group at T1. The gender and the group revealed no significant relationship [χ^2^(1) = 2.234, *p* = 0.208, Φ = 0.168]. The proportion of boys from the completers clinical group was 91.3% (21 boys), while it was 76.8% (43 boys) in the non-completers clinical group at T1. Social status and the group (completers and non-completers) revealed no significant relationship [χ^2^(3) = 5.436, *p* = 0.143, Φ = 0.266].

### MINI Kid Scales and HRQoL in Clinical and Control Group at T1

There were statistically significant differences between the clinical and the control groups in 9 of the examined 13 scales of the MINI Kid at T1, with the exceptions being the scales for panic disorder, specific phobia, obsessive-compulsive disorder, and tic disorder. Children in the clinical group were characterized higher scale scores than the control group (Table 2).

**TABLE 2:** MINI Kid scales in the clinical and the control group at T1.

	**Control**		**Clinical**		***t***	***p***
	***M***	***SD***	***M***	***SD***		
Major depressive episode	5.53	8.98	34.71	26.56	–4.892	< 0.001
Dysthymic disorder	9.66	14.72	44.44	30.09	–4.891	< 0.001
(Hypo)manic episode	10.51	11.01	45.45	26.82	–5.672	< 0.001
Panic disorder	1.37	3.26	11.00	25.98	–1.726	0.099
Attention deficit/hyperactivity disorder	10.87	14.62	73.32	18.50	–12.701	< 0.001
Separation anxiety disorder	9.06	12.29	26.14	32.96	–2.283	0.031
Social phobia	1.09	5.21	35.23	46.07	–3.455	0.002
Specific phobia	18.48	27.40	20.45	36.71	–0.205	0.838
Obsessive compulsive disorder	1.74	8.34	5.45	15.35	–1.003	0.323
Tic disorder	5.80	15.58	9.42	23.48	–0.617	0.541
Conduct disorder	3.48	4.68	28.34	20.35	–5.586	< 0.001
Oppositional defiance disorder	7.83	9.51	65.00	29.24	–8.740	< 0.001
Generalized anxiety disorder	0.48	2.32	19.05	31.26	–2.715	0.013

Significant differences appeared between the clinical and the control group in HRQoL according to both the children [*t*(41) = 3.709, *p* = 0.001] and the parents [*t*(42) = 8.208, *p* < 0.001]. Children in the clinical group showed poorer HRQoL than the control group. There were no significant difference in both self- [*t*(75) = −1.138, *p* = 0.259] and parent-reported [*t*(75) = −0.724, *p* = 0.471] HRQoL and also in ADHD MINI Kid scale [*t*(77) = −0.542, *p* = 0.591] between completers (*M* = 73.32, *SD* = 18.50) and non-completers (*M* = 75.65, *SD* = 14.10) groups at T1.

### Primary Outcomes at T2

#### MINI Kid Scales

**FIGURE 1:**
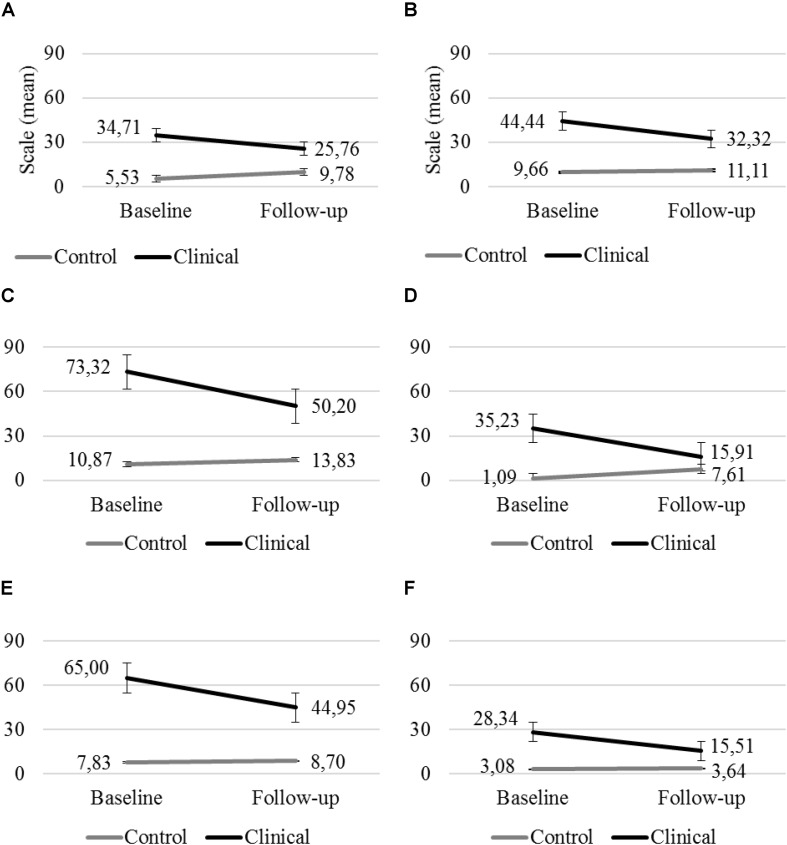
Major depressive episode **(A)** and dysthymic disorder **(B)**, Attention deficit/hyperactivity disorder **(C)**, social phobia **(D)**, oppositional defiance **(E)**, and conduct disorder **(F)** scales in the clinical and the control group at T1 and T2 (error bar: standard error).

Of the 13 MINI Kid scales, 6 scales showed significant Time main effect and/or Time × Group interaction. The scores for these scales indicated statistically significant changes between T1 and T2 independent of Group (clinical vs. control). Scale scores showed significant decreases independent of Group in Time. There was also a significant Time × Group interaction in the scales for major depressive episode and dysthymic disorder, and the change was different in the groups. The scores for both scales were constant or increased in a non-significant way in the control group, whereas these scores decreased significantly in the clinical group (Figure 1).

For the other four scales – attention deficit/hyperactivity disorder, social phobia, oppositional defiance, and conduct disorder – the only significant finding was the Time × Group interaction effect. The scores for these scales were constant or increased in a non-significant way in the control group, whereas they decreased significantly in the clinical group (Figure 1).

There were no significant effects for the remaining seven scales: (hypo)manic episode, panic disorder, separation anxiety disorder, specific phobia, obsessive-compulsive disorder, tic disorder, and generalized anxiety disorder. Based on the means of the scores of these scales, the scores were constant or increased in the control group, whereas they decreased in the clinical group in non-significant way, with the exception of obsessive-compulsive disorder, the scores for which were constant or followed similar patterns in both groups, remaining constant in the control group and increasing non-significantly in the clinical group (Table 3).

**TABLE 3:** MINI Kid scales in the clinical and the control group at T1 and T2.

	**T1 *M* (*SD*)**		**T2 *M* (*SD*)**		**Time**			**Time × Group**		
	**Control**	**Clinical**	**Control**	**Clinical**	***F***	***p***	**${\text{η}}_{\text{p}}^{2}$**	***F***	***p***	**${\text{η}}_{\text{p}}^{2}$**
*Major depressive episode*	*5.53 (8.98)*	*34.71 (26.56)*	*9.78 (15.62)*	*25.76 (24.25)*	*9.719*	*0.003*	*0.19*	*7.293*	*0.010*	*0.15*
*Dysthymic disorder*	*9.66 (14.72)*	*44.44 (30.09)*	*11.11 (12.97)*	*32.32 (21.66)*	*4.550*	*0.039*	*0.10*	*6.434*	*0.015*	*0.13*
(Hypo)manic episode	10.51 (11.01)	45.45 (26.82)	21.38 (25.23)	46.21 (29.06)	0.141	0.709	0.00	1.301	0.261	0.03
Panic disorder	1.44 (3.32)	11.00 (25.98)	5.02 (9.67)	11.24 (14.55)	2.891	0.097	0.06	0.888	0.352	0.02
*Attention deficit/hyperactivity disorder*	10.87 (14.62)	73.32 (18.50)	13.83 (17.79)	50.20 (26.77)	*0.125*	*0.725*	*0.00*	*12.314*	*0.001*	*0.22*
Separation anxiety disorder	8.33 (10.97)	12.88 (23.38)	6.77 (9.24)	9.85 (21.35)	1.610	0.217	0.06	0.044	0.836	0.02
*Social phobia*	1.09 (5.21)	35.23 (40.07)	7.61 (23.15)	15.91 (34.97)	*0.308*	*0.582*	*0.00*	*4.824*	*0.034*	*0.10*
Specific phobia	18.48 (27.40)	20.45 (36.71)	16.30 (23.37)	21.59 (32.09)	1.175	0.285	0.03	0.015	0.903	0.00
Obsessive compulsive disorder	1.74 (8.34)	5.45 (15.35)	4.35 (20.85)	24.24 (38.74)	0.308	0.582	0.00	1.707	0.198	0.04
Tic disorder	5.80 (15.58)	9.42 (23.48)	7.61 (20.55)	9.78 (24.70)	2.636	0.112	0.06	1.647	0.206	0.04
*Conduct disorder*	*3.08 (4.41)*	*28.34 (20.35)*	*3.64 (5.42)*	*15.51 (11.84)*	*0.525*	*0.473*	*0.01*	*11.037*	*0.002*	*0.22*
*Oppositional defiance disorder*	*7.83 (9.51)*	*65.00 (29.24)*	*8.70 (15.00)*	*44.95 (34.71)*	*1.539*	*0.222*	*0.04*	*7.592*	*0.009*	*0.15*
Generalized anxiety disorder	0.48 (2.32)	19.05 (31.26)	11.59 (19.67)	17.99 (23.69)	1.475	0.231	0.04	2.557	0.117	0.06

*SD, standard deviation; F, F-value; p, p-value; $\eta_{p}^{2}$, partial eta squared.*

#### HRQoL According to the Children and Parents

**FIGURE 2:**
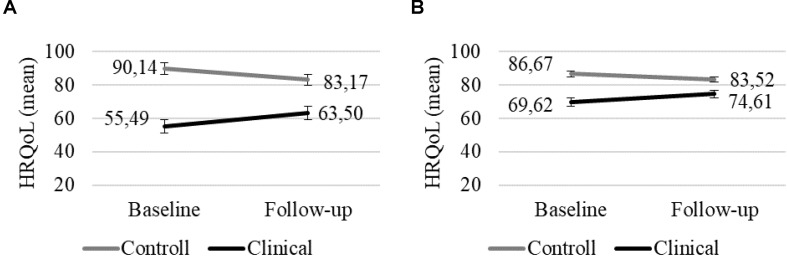
HRQoL according to the parents **(A)** and children **(B)** in the clinical and the control group at T1 and T2 (error bar: standard error).

Health-related quality of life according to the parents indicated statistically significant Time × Group interaction [*F*(1,41) = 11.264, *p* = 0.002, ${\text{η}}_{\text{p}}^{2}$ = 0.22]. HRQoL was significantly higher in the clinical group at T2 than at T1, indicating better HRQoL. In the control group, there were no significant changes, and based on the means, the HRQoL decreased in non-significant way, indicating an unchanged or worse HRQoL. HRQoL according the children showed only a marginally significant Time × Group interaction [*F*(1,38) = 3.570, *p* = 0.066, ${\text{η}}_{\text{p}}^{2}$ = 0.09]. The changes showed a similar pattern to those in the parent-reported HRQoL (Figure 2).

### Secondary Outcomes at T2

#### Gender

First, it is important to emphasize that there were only two girls in the clinical group at T2. There was a statistically significant Group × Gender interaction for the panic disorder [*F*(1,39) = 4.295, *p* = 0.045, ${\text{η}}_{\text{p}}^{2}$ = 0.10] and social phobia [*F*(1,40) = 14.023, *p* = 0.001, ${\text{η}}_{\text{p}}^{2}$ = 0.26] scales, which means that the changes were significantly different in the groups depending on gender.

The scale for panic disorder was constant or its changes were non-significant in the control group for both boys and girls. In the clinical group, it was constant or decreased in a non-significant way for boys, but increased significantly in girls.

The scale for social phobia was constant or increased in a non-significant way in the control group for both boys and girls. In the clinical group, it significantly decreased for both boys and girls, although this decrease was greater for girls than for boys.

Health-related quality of life according to the children and parents resulted non-significant main [children: *F*(1,36) = 0.095, *p* = 0.759; parents: *F*(1,39) = 0.183, *p* = 0.671] or interaction effects [children: *F*(1,36) = 0.300, *p* = 0.587; parents: *F*(1,39) = 0.335, *p* = 0.566] considering gender.

#### Social Status

According to both the children and parents, the 13 scales of the MINI Kid and HRQoL resulted in non-significant main or interaction effects considering social status.

## Discussion

To our knowledge the current study is the first one to investigate the association between multimodal therapy on ADHD and comorbid disorders and HRQoL from both the children’s and parents’ perspectives over the long term. As mentioned above, several studies have established the positive short-term effect of pharmacotherapy on HRQoL and on the remission of ADHD symptoms, but most of these focused on the acute treatment period, and there is a lack of studies regarding the impact of multimodal therapies on HRQoL (Coghill, 2010; Coghill et al., 2017). In addition, the majority of studies did not allow the recruitment of those with co-morbid disorders (Coghill, 2010), despite the fact that the existence of comorbid disorders is more a rule than an exception in ADHD (Thompson et al., 2004). Our study therefore has major clinical implications for assessing the possible effects of therapy with assessment and follow-up on ADHD and comorbid symptoms, as well as on HRQoL.

At baseline, treatment-naïve children with ADHD showed higher psychopathology scores and lower levels of HRQoL than the healthy control group according to both their own and their parents’ judgment. This result is consistent with the findings of other investigations that have established that children with ADHD experience impairment in their HRQoL (Danckaerts et al., 2009; Velõ et al., 2013). Based on the conclusions of these studies, effective treatment of ADHD has to focus not only on the reduction of symptoms but also on the improvement of patients’ HRQoL (Coghill, 2010).

A notable finding of our study is that six symptom scales showed significant long-term changes over time and these changes were different between the groups. The symptoms of ADHD and major depressive disorder, dysthymic disorder, social phobia, oppositional deviant disorder, and conduct disorder were decreased in the clinical group, while at the same time these symptoms were remained constant or showed a non-significant increase in the control group. Incidence of major depressive disorder and dysthymic disorder in the control group appeared to be rising as these subjects approached adolescence (Costello et al., 2003; Nobile et al., 2003; Thompson et al., 2004), but our results showed a decrease over time in the clinical group. These findings could be due to the multimodal treatment applied in the clinical group. It may reflect to the floor effect, that there was no significant change in the control group; however, the control group could have shown natural change in time too.

The other important finding was an improvement in HRQoL in the clinical group from the parents’ perspective, although there was a slightly negative but non-significant change in the control group. Based on the evaluation by the children, there was only a marginally positive change in HRQoL in the clinical group. We described above, that previous studies established, lower parent-reported HRQoL was related to older age (Dallos et al., 2017), and as it increases over time in the present study, it may suggest positive interpretation regarding the effectiveness of multimodal treatment. Further studies on a larger sample are needed to evaluate which domains of HRQoL are responsible for this result, as well as the special intervention needs to be based on these HRQoL domains.

Our findings revealed that changes in ADHD and comorbid symptoms and HRQoL may be independent of gender and social status. In the case of two scales (panic disorder and social phobic disorder) there was a gender effect statistically, but we cannot draw a conclusion based on these results because only two girls were in the clinical sample at follow-up.

There are several limitations of the present study which must be acknowledged. The study focuses only on children who were able to participate in study for 36 months. Our result cannot be extended to that population who did not participate in the multimodal treatment for 36 months. It can give partly explanation of our second limitation: the low sample size. During such a long-term program, many children passed out of the care system, having changed residence, not requiring further treatment or just did not want to participate in the extra visit. However, we compared the completers and non-completers groups in several domains at baseline, such as age, gender, social status, self- and parent-reported HRQoL, and symptom severity, and we found no significant difference between them. The third limitation of the study we have to add that due to ethical reason we could not randomized treatment-naïve children with ADHD further into two groups, i.e., (1) treatment-naïve children with ADHD who get further treatment for long duration and (2) treatment-naïve children with ADHD, who do not get further treatment for long duration. We included all children into the previously treatment-naïve group, whose further combined treatment was administered according to the instructions of their child psychiatrist in the hospital. Fourth, there was a gender difference between the clinical and control groups, although this does reflect the experience of clinical practice as well, because ADHD is more common among boys (Pastor et al., 2011); the gender ratio was approximately equal in the control sample as in non-clinical, Hungarian population (Hungary, 2016). Fifth, although the MINI Kid evaluates a wide range of child and adolescent *DSM-IV* psychiatric diagnoses, it is also important to assess other comorbid disorders, such as learning disorders that are present in almost 25% of ADHD cases (Pliszka, 1998). Sixth, we did not assess the physical status and family structure of the children, which may also have an impact on HRQoL as well. Seventh, despite the fact that parents participated in parental training and had half-yearly consultations with professionals about the effective use of behavioral interventions, our investigation did not cover whether parents actually and consistently applied these interventions with their children. This study result is valid for those children who could be participated for this study 36 months (i.e., lack of side effects and having the necessary adherence to the therapy). Further interpreting our results we should take into consideration that other factors (i.e., family operation) may lead to improvements in the HRQoL of children with ADHD not only multimodal treatment.

Despite these limitations, the current study provides much valuable information to practitioners. Most of the studies in this field have demonstrated the short-term effects of ADHD therapy, while our aim was to evaluate the possible long-term effects, which is more relevant due to the persistence of the disease over time (Brown et al., 2001; Biederman et al., 2010; Polanczyk et al., 2014). The present study highlights not only the effect of medication, but also the effect of the combination of medication and behavioral interventions. In addition to ADHD, we also give information on the comorbid disorders, which can play a significant role in the patient’s HRQoL. Finally, our study measures the disorders using a dimensional approach, which adds more information about the possible effects of the therapy.

## Conclusion

In conclusion, our study highlights that multimodal therapy has beneficial long-term effects not only in the decrease of ADHD symptoms, but also in the relief of the comorbid disorders. These positive effects are also reflected in the changes in the HRQoL of patients, which was perceived by both the parents and children. Although the questionnaire methods provide reliable information about HRQoL, the experienced changes as a result of treatment cannot be precisely identified from the children’s perspective. In assessing the HRQoL, for further research should therefore consider the use of qualitative interviews, with could provide much more detailed information about the effects of therapy on the HRQoL of children with ADHD.

## Data Availability

The datasets generated for this study are available on request to the corresponding author.

## Ethics Statement

The studies involving human participants were reviewed and approved by the Ethical Committee of the Medical Research Council, Hungary (ETT-TUKEB). Written informed consent to participate in this study was provided by the participants’ legal guardian/next of kin.

## Author Contributions

ÁK and GF-D participated in the data collection. SV and ÁK coordinated the implementation and the data entry. SV participated in the data collection and in the statistical analyses. GF-D participated in the implementation. JB ideated and designed the study and trained the personnel. SV, ÁK, GF-D, and JB jointly drafted the manuscript.

## Conflict of Interest Statement

The authors declare that the research was conducted in the absence of any commercial or financial relationships that could be construed as a potential conflict of interest.
